# Ultrafast Fabrication of Flexible Dye-Sensitized Solar Cells by Ultrasonic Spray-Coating Technology

**DOI:** 10.1038/srep14645

**Published:** 2015-09-30

**Authors:** Hyun-Gyu Han, Hashitha C. Weerasinghe, Kwang Min Kim, Jeong Soo Kim, Yi-Bing Cheng, David J. Jones, Andrew B. Holmes, Tae-Hyuk Kwon

**Affiliations:** 1 Department of Chemistry, School of Natural Science, Ulsan National Institute of Science and Technology, Ulsan, 689-798, Republic of Korea; 2 Commonwealth Scientific and Industrial Research Organisation (CSIRO), Melbourne, Victoria 3169, Australia; 3 Department of Materials Engineering, Monash University, Clayton, Victoria 3800, Australia; 4 School of Chemistry, Bio21 Institute, University of Melbourne, Parkville, Victoria, 3010, Australia

## Abstract

This study investigates novel deposition techniques for the preparation of TiO_2_ electrodes for use in flexible dye-sensitized solar cells. These proposed new methods, namely pre-dye-coating and codeposition ultrasonic spraying, eliminate the conventional need for time-consuming processes such as dye soaking and high-temperature sintering. Power conversion efficiencies of over 4.0% were achieved with electrodes prepared on flexible polymer substrates using this new deposition technology and N719 dye as a sensitizer.

Flexible dye-sensitized solar cells (DSC) have received considerable attention for use in a wide range of potential commercial applications, such as indoor or mobile devices, as well as for building integrated photovoltaic modules^1^,^2^,^3^,^4^,^5^,^6^,^7^. This can be mainly attributed to the fact that the spectrum of the different dyes used in DSCs fits well with that of indoor lightning sources, as well as indirect sunlight diffused through windows. Indoor devices such as remote sensors and alarm systems using indoor lighting conditions in particular requires a very low power density (0.5–1 mW/cm^2^) that can be obtained with power conversion efficiency (PCE) of less than 3%^8^. Moreover, the device performance of transparent DSCs can be greater than that of competing transparent amorphous silicon solar cells^9^.

Despite these advantages, current fabrication processes for DSCs have intrinsic barriers that hinder their use in the continuous mass production of DSC modules using speedy roll-to-roll methods. The first of these is the time-consuming nature of the dye-soaking process, which results from the need to create a high density of dye molecules attached to the surface of TiO_2_ nano-particles. Furthermore, this lengthy dye soaking process provides no control over the amount of dye adsorbed on TiO_2_ electrodes, which has important implications with regards to device performance and reproducibility. However, as far as we can tell, there have been no reports so far of a dye-soaking process that does allow control over the amount of dye on the TiO_2_ surface, nor is there an alternative dye soaking method available that is fast enough to be compatible with speedy roll to roll fabrication. The other critical issue preventing mass production is that the high-temperature (>450 °C) of the sintering process needed to enhance electrical contact between TiO_2_ nanopowder particles (through so-called necking of nano-particles), as well as remove residual solvents and organic binders, is not compatible with flexible polymer substrates due to their thermal degradation at temperatures above 150 °C. Most efforts toward fabricating flexible DSCs have therefore been focused on enhancing the connectivity of TiO_2_ electrodes under low-temperature conditions (≤150 °C)^2^,^3^,^6^,^10^,^11^,^12^,^13^. Such research is, however, still far from achieving speedy device fabrication, as it does not eliminate the need for a time-consuming soaking process.

To overcome these problems, this study investigates the newly developed deposition techniques of pre-dye-coating and codeposition ultrasonic spraying. These new technologies allow for the ultrafast preparation of dye-coated TiO_2_ electrodes on flexible polymer substrates, thereby avoiding the need for a lengthy dye-soaking process. Following the deposition of dye-coated TiO_2_ films onto flexible substrates using either technique, cold isostatic pressing (CIP) was used in place of conventional high-temperature sintering to prepare mechanically robust TiO_2_ electrodes with an enhanced inter-particle connectivity between dye-coated TiO_2_ particles. This process was selected on the basis that the morphology and physical properties of TiO_2_ electrodes prepared by CIP are known to be suitable for use with flexible DSCs on plastic substrates prepared without the addition of an organic binder or heat treatment^4^. In addition, the pressed TiO_2_ electrodes become more transparent and therefore increase light transmission^4^. Despite eliminating lengthy dye-soaking and high-temperature sintering processes, this simple and rapid spray process still allows for the fabrication of flexible DSC with a power conversion efficiency of over 4.0%. These new technologies therefore represent a significant step towards achieving the speedy fabrication of flexible DSC modules for indoor applications that do not require a high power density.

## Results

**Figure 1:**
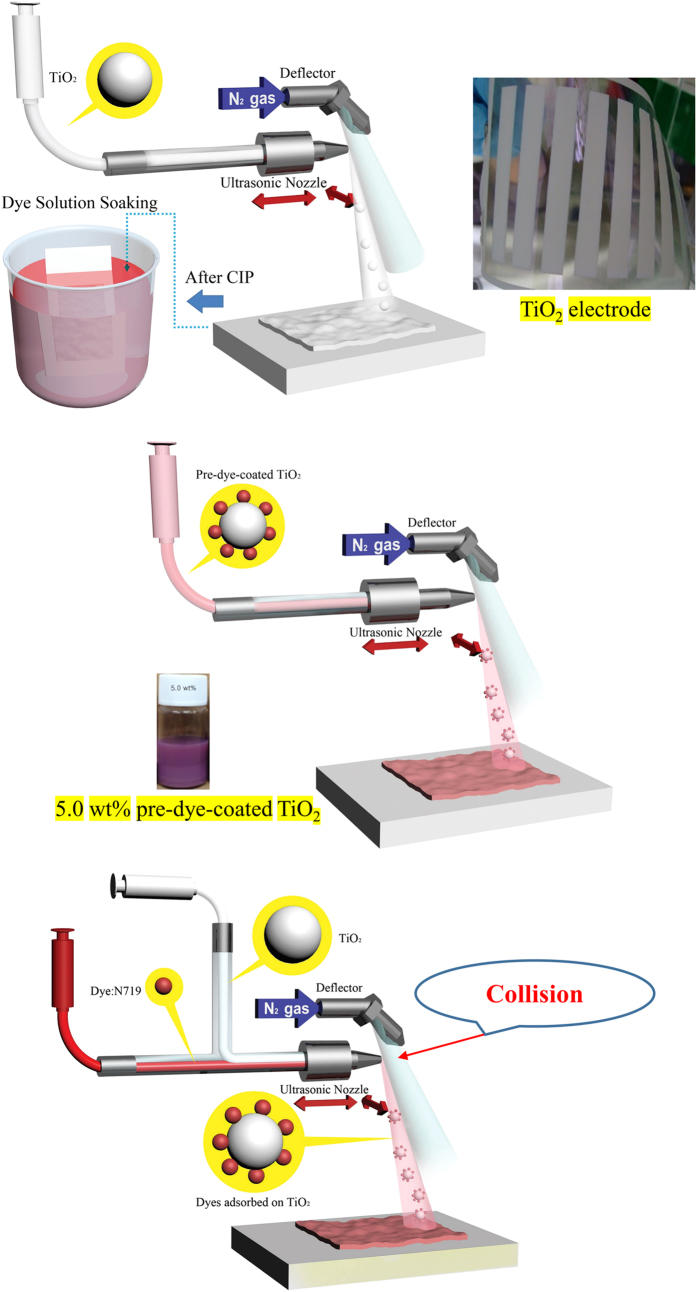
Schematic showing the fabrication of flexible DSCs using a combination of dye soaking and ultrasonic spray technology, pre-dye-coating ultrasonic spray technology, and codeposition ultrasonic spray technology. In all instances, TiO_2_ electrodes were prepared by ultrasonic spray coating and cold isostatic pressing (CIP).

Figure 1 shows schematic diagrams of the proposed pre-dye-coating ultrasonic spray and codeposition ultrasonic spray technologies, as well as the conventional dye-soaking process. In the case of the former, pre-dye-coated TiO_2_ nanopowder suspensions are first prepared by adding a N719 dye solution to P-25 TiO_2_ nanopowder suspensions (from Degussa), and then sprayed onto an indium tin oxide-coated polyethylene naphthalate (ITO-PEN) flexible substrate to produce a uniformly dye-coated TiO_2_ film. With the codeposition process, a pre-prepared dye solution and P-25 TiO_2_ nanopowder suspension are first placed into individual syringe pumps. These are then forced to collide at end of an ultrasonic nozzle prior to being sprayed onto ITO-PEN, thereby forming dye-coated TiO_2_ electrodes. As can be seen, these new technologies do not require an additional dye soaking procedure or any other complicated TiO_2_ electrode preparation techniques. Furthermore, the amount of dye on the TiO_2_ electrode can be easily controlled by changing the concentration of the dye used for pre-dye-coating and/or the injection rate of the dye solution used for spray codeposition. Using these methods, we have succeeded in readily fabricating flexible TiO_2_ electrodes on large areas of ITO-PEN (10 cm × 1 cm) within a matter of less than twenty minutes, while also accurately controlling the amount of dye on the TiO_2_ particles. For CIP compression, the as-deposited TiO_2_ electrodes were first sealed in a plastic bag using a vacuum sealer, and were then compressed at 200 MPa for 10 min to yield flexible TiO_2_ electrodes containing electrically well-connected TiO_2_ nano-particles. The thickness of the titania film was also found to be dramatically reduced to as much as 66% of its original thickness. The performance of each of the flexible DSC devices was evaluated using N719 dyes under open-cell conditions.

### Conventional soaking deposition

For the purposes of comparison, flexible TiO_2_ electrodes were also prepared using by the conventional approach of spray-coating and CIP, followed by a lengthy dye soaking process. These flexible TiO_2_ electrodes (10 cm × 1 cm) were conventionally fabricated by injecting a solution of 2.5 wt% TiO_2_ (P-25) in EtOH at a rate of 0.2 ml/min onto an ITO-PEN substrate heated to 100 °C (Fig. 1(a)) and were then immersed overnight (<12 hours) in a solution of 0.1 mM N719 in ethanol to produce dye-coated TiO_2_ electrodes. Next, flexible DSC devices were constructed by sandwiching a Pt/ITO-PEN counter electrode with pre-dye-coated TiO_2_/ITO-PEN, and then infiltrating it with an electrolyte composed of 0.04 M I_2_, 0.4 M tetrabutylammonium iodide, 0.4 M lithium iodide, and 0.3 M *N*-methylbenzimidazole in acetonitrile and 3-methoxypropionitrile (1:1 ratio by volume). The resulting device performance was measured under standard conditions (1 sun), and the results obtained are summarized in Table 1. For a titania film thickness of 8 μm, the maximum power conversion efficiency (PCE) was determined to be 4.7% with a *J*_sc_ of 10.9 mA/cm^2^, *V*_oc_ of 736 m*V*, and *ff* of 0.59 (Figure S1).

### Pre-dye-coating deposition

Figure 1(b) shows a schematic diagram of the pre-dye-coated deposition technique, which requires a low-viscosity TiO_2_ paste without any organic binder (~2.5 wt% relative to the solvent weight) in EtOH in order to allow easy injection through a syringe pump. To determine the optimum dye:TiO_2_ ratio, various P-25 TiO_2_ nanopowder suspensions were prepared, each coated with a different concentration of N719 dye (2.5–10 wt% relative to the weight of TiO_2_). The change in the white colour of the P-25 TiO_2_ to pink that is evident in Figure S2 occurred within just a few seconds of adding the N719 dye solution, which is indicative of a near-instant adsorption of the dye molecules. Only a 5.0 wt% solution of dyes was homogenously saturated into the TiO_2_ nano-powder particles without any dye remaining in the supernatant and the resulting device performance was the best of various dye:TiO_2_ ratio. (full details are provided in the supporting information, Figure S2) The level of dye adsorption observed with 5 wt% dye-coated TiO_2_ corresponds to 88% of that achieved through conventional soaking (see Table S1), but this slightly less dye adsorption has little effect on the current density. Similar results are also achieved through codeposition (Table 1). Thus, using pre-dye-coated deposition allows for an optimized amount of dye and reproducibility to be easily controlled.

**Figure 2:**
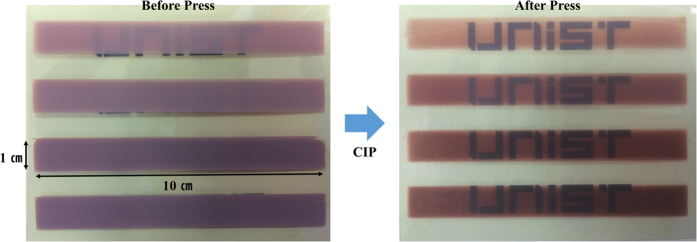
TiO_2_ electrodes fabricated by pre-dye-coating spray technology with different deposition layers shown before cold isostatic pressing and after cold isostatic pressing.

**Figure 3:**
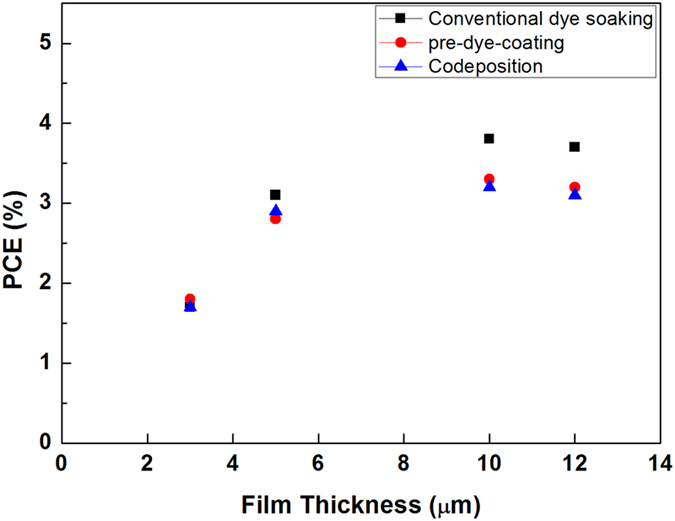
Photo conversion efficiency (PCE) vs. film thickness of the dye-coated TiO_2_ electrodes by conventional dye soaking, pre-dye-coating and codeposition processes.

**Figure 4:**
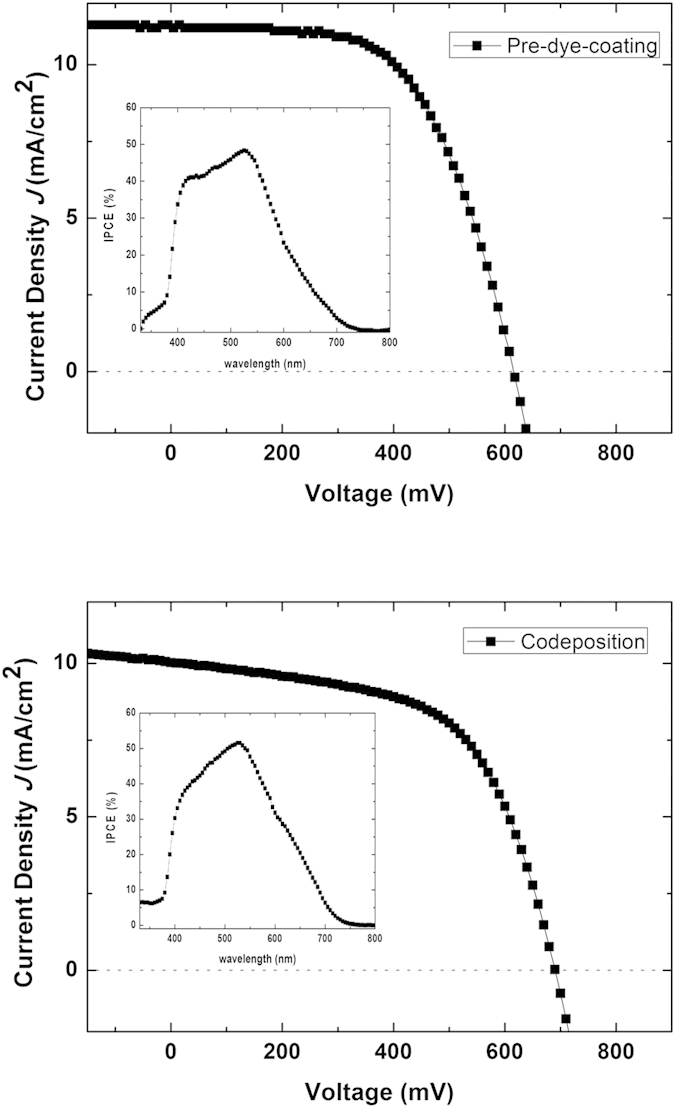
*I*–*V* characteristics of a flexible DSC prepared using pre-dye-coating spray technology with N719 dye on TiO_2_ and codeposition spray technology with N719 dye on TiO_2_ electrodes. Each inset shows the incident-photon-to-current conversion efficiency.

To optimize and control the film thickness produced by ultrasonic spray coating deposition, electrodes of different thicknesses were prepared by changing the number of deposition layers. Following CIP compression, this resulted in values of 2, 4, 6, 8, 10, and 18 μm. The structure of these isostatically compressed electrodes was found, through scanning electron microscopy (SEM) to be highly compact, and much unlike the unpressed films (Figure S3). In addition, the images of the dye-coated electrodes before and after CIP shown in Fig. 2 reveal that the transparency of the TiO_2_ electrodes is enhanced by CIP, which is consistent with previously reported results^4^. This is also true in the case of the inter-particle connectivity between TiO_2_ nanoparticles, as evident in the electrodes prepared by codeposition (Figure S4). The PCE of the aforementioned DSC devices was found to increase from 2 to 3.4% when the TiO_2_ film thickness was increased from 3 to 10 μm, but decreased beyond this point, suggesting that the optimal film thickness lies somewhere within the range of 8–10 μm. Similar results are also observed with both conventional soaking and codeposition processes in Fig. 3. An optimized DSC device with pre-dye-coated TiO_2_ electrodes resulted in a PCE of 4.1% at an open-circuit voltage (*V*_oc_) of 620 mV, short-circuit current (*J*_sc_) of 11.2 mA/cm^2^, and *ff* of 0.58. Figure 4 shows the corresponding *I*–*V* characteristics of this optimized device, with its incident photon-to-current conversion efficiency (IPCE) spectra shown in Fig. 4(a) inset. Note that its maximum IPCE reaches 48% at a wavelength of 525 nm.

### Codeposition process

As shown in the schematic diagram in Fig. 1(c), this method entails injecting separate solutions of N719 dye and a 5 wt% P-25 TiO_2_ suspension into an ultrasonic nozzle to obtain a constant dye concentration of 2.5 wt% TiO_2_ in ethanol; that is, the same optimized concentration as was used for the pre-dye-coating processes. The level of dye adsorption by codeposition process corresponds to 73% that achieved through the conventional soaking process (see Table S1). By controlling the injection rate to further optimize the process, flexible dye-coated TiO_2_ electrodes were fabricated onto ITO-PEN substrates heated to 100 °C. These were then isostatically pressed to a final thickness of 8 μm, resulting in a highly compact structure that was again confirmed by SEM imaging (Figure S3).

Sandwiched DSC devices were fabricated using the same procedure as that used with the electrodes fabricated by pre-dye-coating, and their *I*–*V* characteristics are summarized in Table 1. This shows that an optimized device fabricated using codeposition ultrasonic spray coating exhibits a *J*_sc_ of 10.0 mA/cm^2^, *V*_oc_ of 690 mV, and *ff* of 0.58, resulting in an overall PCE of 4.0%. The corresponding *I*–*V* curves and IPCE are shown in Fig. 4(b) and its inset, respectively. Notably, these results are comparable to those obtained using pre-dye-coating ultrasonic spray technology, with the maximum IPCE reaching 52% at a wavelength of 525 nm. However, when compared with the device performance achieved with the conventional soaking process, both the pre-dye-coating and codeposition processes produce a slightly lower performance due mainly to the decrease in *V*_oc_, while the current density and fill factor remain at a similar or higher value. The reason for this is not clear but can be assumed to be related to the exposure of the dye-coated TiO_2_ electrodes to ambient air and light during the device fabrication process and/or the open cell device structure.

### Binding mode and dye stability after CIP compression

**Figure 5:**
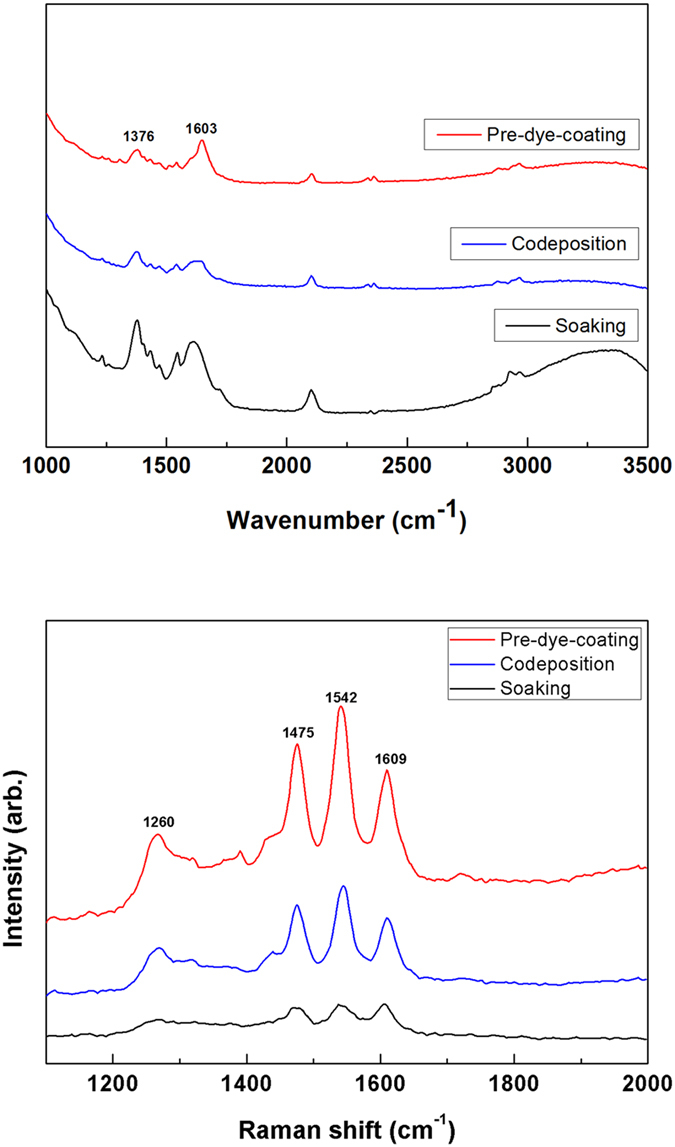
FT-IR and Raman spectrum of N719 adsorbed on TiO_2_ electrodes prepared by conventional soaking (black) or codeposition (blue) and pre-dye-coating (red) processes, and then subjected to CIP compression for.

The notion of whether these newly developed ultrafast fabrication processes for dye-coated TiO_2_ electrodes produce a chemical adsorption comparable to the conventional soaking process was investigated, as the binding mode of the dye molecules plays an important role in ensuring that excited electrons are efficiently injected from the dye molecule into the TiO_2_ particles. Specifically, there are three different binding modes that can possibly exist when the carboxylate group of a dye coordinates to a TiO_2_ surface: a unidentate mode, a chelating mode, and a bridging bidentate mode^14^. Note that it is the latter of these that is preferred if the aim is to a high quantum yield for electron injection into the TiO_2_ Fermi level. The binding mode associated with dye adsorption on a TiO_2_ surface can be determined by calculating the frequency separation between the asymmetric and symmetric stretching modes of the carboxylate unit through resonance Fourier transform infrared (FT-IR) analysis^14^. This method was applied to the dye-coated TiO_2_ electrodes prepared by conventional soaking, pre-dye-coating and codeposition processes, and the FT-IR spectra obtained are shown in Fig. 5(a). From this, we can see that asymmetric and symmetric stretching bands appear at 1603 cm^−1^ and 1376 cm^−1^, respectively, for the carboxylate of the N719 dye adsorbed on the TiO_2_ surface prepared by conventional soaking, which corresponds to a bidentate mode. Similar FT-IR results were obtained for the N719 dye on the TiO_2_ surfaces prepared by pre-dye-coating (red) and spray codeposition (blue); however, the distinct peak evident at around 1720 cm^−1^ in the FT-IR spectrum of the dried N719 dye powder is notably absent^14^,^15^. This indicates that N719 dye molecules are indeed chemically adsorbed on the TiO_2_ surface when pre-dye-coating or codeposition ultrasonic spray technology is applied. These dye-coated samples were further characterized using Raman spectroscopy, but again no distinguishable difference was observed in their spectra (Fig. 5(b)). Instead, all samples exhibited distinct peaks at 1260, 1475, 1542 and 1609 cm^−1^ that are attributed to vibrational modes of the 4,4′-dicarboxy-2,2′-bipyridine ligand^15^. On the basis of this, it is concluded that ultrasonic spray technology achieves a very similar binding mode of dye molecules on a TiO_2_ surface as conventional soaking^15^.

**Figure 6:**
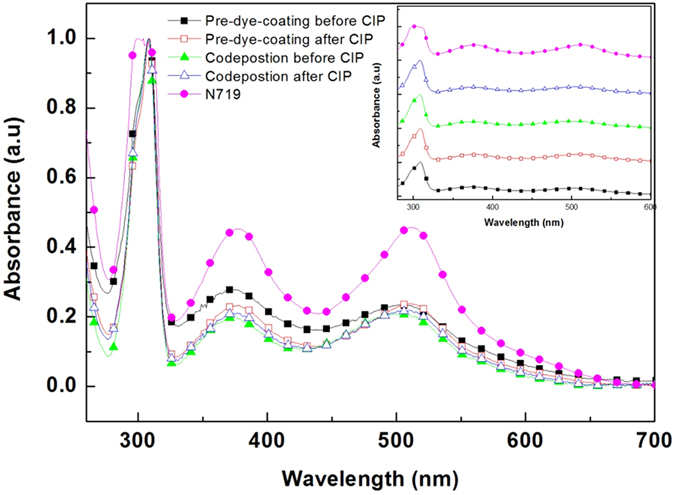
UV-vis absorption spectrum of detached N719 dyes from a pre-dye-coating solution prior to CIP (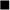) and after CIP (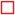), a codeposition solution prior to CIP (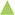) and after CIP (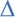), and a N719 reference (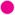) after normalization. The inset shows the respective UV-vis spectrum for each.

**Figure 7:**
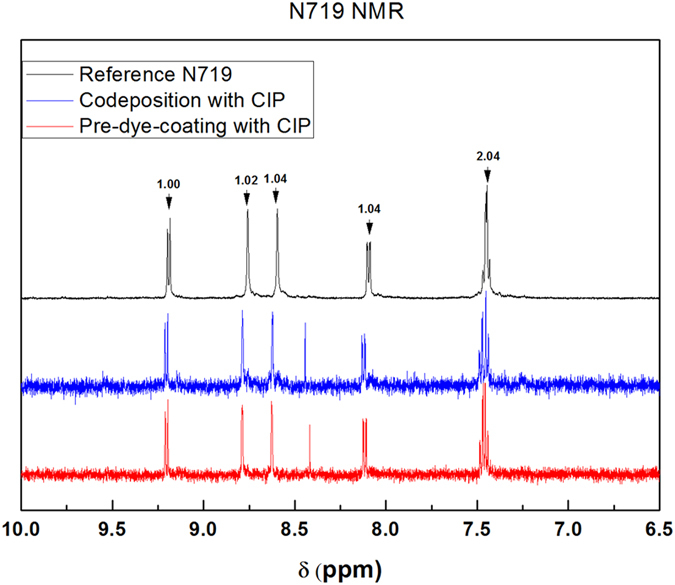
^1^H NMR spectra for the aromatic region of N719 dye obtained from solutions of N719 (black), codepositon with CIP (blue), and pre-dye-coating with CIP (red) in DMSO-d_6_ containing 0.1 M NaOD.

The possibility of dye decomposition during CIP was investigated through ultraviolet-visible (UV-vis) and nuclear magnetic resonance (NMR) spectroscopy. For the UV-Vis investigation, four different types of detached N719 dye solutions were prepared by desorbing with a 0.1 M KOH ethanol solution a: pre-dye-coating solution prior to CIP, pre-dye-coating solution after CIP, codeposition solution before CIP and codeposition solution after CIP (details of this are provided in the supporting information). To provide a suitable reference, N719 dye in an ethanol:water (1:1 v/v %) solution containing 0.1 M KOH was also prepared. Comparison of the normalized UV-vis spectra of these solutions (Fig. 6) shows that they are all very similar to each other, with two broad metal-to-ligand charge transfer (MLCT) bands at 370 nm and 507 nm, and an intraligand (π-π*) charge-transfer transition at 307 nm. For the NMR investigation, along with a N719 dye reference, two different types of detached N719 dyes were prepared through desorption with 0.1 M NaOH: codeposition with CIP and pre-dye-coating with CIP (see the supporting information for details). As can be seen in Fig. 7, the NMR results obtained from deuterated dimethyl sulfoxide (DMSO-*d*_6_) show no difference between the reference N719 and either codeposition with CIP or pre-dye-coating with CIP solutions. As a result, it can be safely concluded that there is no decomposition of the dye during CIP.

## Discussion

By using the newly developed and rapid pre-dye-coating or codeposition ultrasonic spray-coating techniques in conjunction with CIP, we have succeeded in fabricating flexible DSC devices without requiring conventional dye soaking or high-temperature sintering processes and easily controlled the amount of dye-adsorption on the TiO_2_ surface. Furthermore, dye-coated electrodes were fabricated at a large scale (10 cm × 1 cm) within a very short period of time (<20 minutes). A PCE of 4.1% was obtained using pre-dye-coating ultrasonic spray coating, and 4.0% was obtained using codeposition ultrasonic spray technology, which corresponds to 85% of the 4.7% PCE obtained using a conventional soaking process. Although the device performances achieved with these new technologies is low compared with using state-of-the-art device architecture, the advantages of speedy device fabrication may prove a very useful and cost effective tool for indoor devices that do not require a high power density. Moreover, further optimization of the device fabrication conditions, such as employing an inert and/or low light atmosphere and fully sealing the cell, could conceivably increase its performance significantly. In addition, this concept may prove useful for fabricating multiple TiO_2_ layers with compensating absorption ranges to provide panchromatic absorption. These proposed new technologies are therefore believed to represent a new breakthrough in the fabrication of flexible DSC devices, particularly indoor devices, and could be readily adapted to the mass production of large-area devices in the near future.

In summery this study highlights the development of new electrode deposition methods for the preparation of efficient DSCs on flexible polymer substrates. Thus the findings are of considerable practical importance for the design of large-scale, roll-to-roll manufacture of printed flexible DSC modules.

## Method

### Ultrasonic spray instruments

To ensure the homogenous deposition of nanosized particles, ultrasonic spray instruments were used in the preparation of all TiO_2_ electrodes used in this study. Specifically, an ultrasonic atomizing nozzle on the spray unit was used to control a jet of nitrogen gas from a flat-jet nitrogen gas deflector, with this immediately entraining the ultrasonically produced spray at the atomizing surface to create a conically shaped spray pattern. The size of the droplets in this spray was governed by the frequency at which the nozzle vibrates (120 kHz in this instance), as well as by the surface tension and density of the liquid being atomized. During this process, an *x*-*y* positioning stage was moved at a constant speed to achieve homogeneous deposition on the substrate. The thickness of the film layer produced could be precisely controlled from tens of nanometers to a few hundred micrometers simply by varying the deposition time, the injection rate of the syringe pump, and the solid loading of the TiO_2_ paste.

### Conventional soaking deposition

Suspensions of ~2.50 wt% TiO_2_ were prepared by dispersing 0.658 g of P-25 TiO_2_ powder in 25.0 g (ca. 32 ml) of 99.7% ethanol. These suspensions were injected at a flow rate 0.2 ml/min by a nitrogen carrier gas under 3 psi of pressure, being then sprayed onto an ITO-PEN substrate (10 cm × 1 cm) with a shadow mask at 100 ˚C. A moveable stage with a horizontal speed of 50 mm/min and spray line space of 1 mm was used to ensure a homogenous spray over the entire surface (10 cm × 1 cm). The resulting TiO_2_ electrodes were transferred to a polyethylene envelope and sealed under a vacuum of 10^−1^ Torr, then pressed for 10 min at room temperature using a cold isostatic pressure (CIP) instrument. The pressure used was varied by 100 Mpa between samples, with all electrodes being then immersed in a 0.1 mM N719 solution (Purchased from Solaronix, Switzerland) in ethanol overnight at room temperature to give dye-coated TiO_2_/ITO-PEN.

### Pre-dye-coating deposition

Titania suspensions were prepared using the same method as for conventional soaking deposition, with the exception of adding 33.0 mg of N719. Spray conditions also remained the same, but the process was repeated until the TiO_2_ electrodes reached the desired thickness. The vacuum-sealed TiO_2_ films were then pressed at room temperature using a cold isostatic pressure (CIP) instrument at 200 Mpa for 10 min.

### Codeposition

Titania suspensions of 5.00 wt% were prepared by dispersing 1.32 g of P-25 TiO_2_ powder (Degussa, Hanau, Germany) in 25.0 g (ca. 31.7 ml) of 99.7% ethanol. A separate dye solution was also prepared by dissolving 33.0 mg of N719 in 25.0 g (ca. 32.0 ml) of 99.7% ethanol, with each solution then being placed into its own separate syringe. These solutions were then injected at a flow rate of 0.1 ml/min by a nitrogen carrier gas under 3 psi pressure, with their collision causing the dye to immediately adsorb onto the TiO_2_ prior to being sprayed through an ultrasonic nozzle onto a ITO-PEN substrate with a shadow mask at 100 °C. This co-deposition spray process was repeated under the same conditions as described above until the desired thickness was obtained. Finally, the vacuum-sealed TiO_2_ films were pressed at room temperature using a cold isostatic pressure (CIP) instrument at 200 Mpa for 10 min.

## Additional Information

**How to cite this article**: Han, H.-G. *et al*. Ultrafast Fabrication of Flexible Dye-Sensitized Solar Cells by Ultrasonic Spray-Coating Technology. *Sci. Rep*. **5**, 14645; doi: 10.1038/srep14645 (2015).

## Supplementary Material

Supplementary Information

## Figures and Tables

**Table 1:** Photovoltaic performance of TiO_2_ electrodes fabricated by spray deposition combined with either conventional dye soaking, pre-dye-coating, or codeposition (0.16 mm^2^ active layer).

Method	Film thickness (μm)	*V*_oc_ (mV)	*J*_sc_ (mA/cm^2^)	*ff*	PCE (%)
Conventional dye soaking	8	726 ± 0.10	10.7 ± 0.02	0.55 ± 0.04	4.3 ± 0.4
Pre-dye-coating	8	610 ± 10.0	10.6 ± 0.6	0.60 ± 0.15	4.0 ± 0.2
Codeposition	8	670 ± 20.0	10.0 ± 0.1	0.58 ± 0.05	3.9 ± 0.2

## References

[b1] Park N. G. . Chemical sintering of nanoparticles: A methodology for low-temperature fabrication of dye-sensitized TiO_2_ films. Adv. Mater. 17, 2349–2353, doi: 10.1002/adma.200500288 (2005).

[b2] Li Y. . Highly durable and flexible dye-sensitized solar cells fabricated on plastic substrates: PVDF-nanofiber-reinforced TiO_2_ photoelectrodes. Energh Environ. Sci 5, 8950–8957, doi: 10.1039/c2ee21674d (2012).

[b3] Weerasinghe H. C., Franks G. V., Plessis J. D., Simon G. P. & Cheng Y. B. Anomalous rheological behavior in chemically modified TiO_2_ colloidal pastes prepared for flexible dye-sensitized solar cells. J. Mater. Chem. 20, 9954–9961, doi: 10.1039/c0jm02063j (2010).

[b4] Weerasinghe H. C., Sirimanne P. M., Simon G. P. & Cheng Y. B. Cold isostatic pressing technique for producing highly efficient flexible dye-sensitised solar cells on plastic substrates. Progress in Photovoltaics 20, 321–332, doi: 10.1002/pip.1140 (2012).

[b5] Lee K. M. . Efficient and stable plastic dye-sensitized solar cells based on a high light-harvesting ruthenium sensitizer. J. Mater. Chem. 19, 5009–5015, doi: 10.1039/b903852c (2009).

[b6] Miyasaka T. & Kijitori Y. Low-temperature fabrication of dye-sensitized plastic electrodes by electrophoretic preparation of mesoporous TiO_2_ layers. J. Electrochem. Soc. 151, A1767–A1773, doi: 10.1149/1.1796931 (2004).

[b7] Heng L. P. . p-n-Junction-Based Flexible Dye-Sensitized Solar Cells. Adv. Funct. Mater. 20, 266–271, doi: 10.1002/adfm.200901671 (2010).

[b8] Nasiri A., Zabalawi S. A. & Mandic G. Indoor Power Harvesting Using Photovoltaic Cells for Low-Power Applications. Ieee Transactions on Industrial Electronics 56, 4502–4509, doi: 10.1109/tie.2009.2020703 (2009).

[b9] Gratzel M. The advent of mesoscopic injection solar cells. Progress in Photovoltaics 14, 429–442, doi: 10.1002/pip.712 (2006).

[b10] Yum J. H., Kim S. S., Kim D. Y. & Sung Y. E. Electrophoretically deposited TiO_2_ photo-electrodes for use in flexible dye-sensitized solar cells. J. Photochem. Photobiol., A 173, 1–6, doi: 10.1016/j.jphotochem.2004.12.023 (2005).

[b11] Durr M. . Low-temperature fabrication of dye-sensitized solar cells by transfer of composite porous layers. Nature. Mater. 4, 607–611, doi: 10.1038/nmat1433 (2005). 16041379

[b12] Zhang D. S., Yoshida T. & Minoura H. Low-temperature fabrication of efficient porous titania photoelectrodes by hydrothermal crystallization at the solid/gas interface. Adv. Mater. 15, 814–817, doi: 10.1002/adma.200304561 (2003).

[b13] Rao A. R. & Dutta V. Achievement of 4.7% conversion efficiency in ZnO dye-sensitized solar cells fabricated by spray deposition using hydrothermally synthesized nanoparticles. Nanotechnology 19, 445712, doi: 10.1088/0957-4484/19/44/445712 (2008). 21832754

[b14] Tian H. N. . Effect of different dye baths and dye-structures on the performance of dye-sensitized solar cells based on triphenylamine dyes. J. Phys. Chem. C 112, 11023–11033, doi: 10.1021/jp800953s (2008).

[b15] Nazeeruddin M. K. . Acid-base equilibria of (2,2 ′-bipyridyl-4,4 ′-dicarboxylic acid)ruthenium(II) complexes and the effect of protonation on charge-transfer sensitization of nanocrystalline titania. Inorg. Chem. 38, 6298–6305, doi: 10.1021/ic990916a (1999). 11671348

