# Supplementary material for: Ultrafast Fabrication of Flexible Dye-Sensitized Solar Cells by Ultrasonic Spray-Coating Technology
Source: Sci Rep. 2015 Sep 30;5:14645. doi: 10.1038/srep14645 (PMC4588502; doi:10.1038/srep14645)
Supplement: Supplementary Information [file srep14645-s1.pdf]

Supporting Information

Ultrafast Fabrication of Flexible Dye-Sensitized Solar Cells by Ultrasonic Spray-Coating Technology

Hyun-Gyu Han,[a] Hasitha C. Weerasinghe,[b, c] Kwang Min Kim,[a,] Jeong Soo Kim, [a] Yi-Bing Cheng, [d] David J. Jones, [b] Andrew B. Holmes, [b, c] Tae-Hyuk Kwon * [a,]

[a]Department of Chemistry, School of Natural Science, Ulsan National Institute of Science and Technology (UNIST), Ulsan, 689-798, Republic of Korea. E-mail:kwon90@unist.ac.kr.

[b]School of Chemistry, Bio21 Institute, University of Melbourne, Parkville, Victoria, 3010, Australia..

[c]Department of Materials Engineering, Monash University, Clayton, Victoria 3800, Australia.

[d]Commonwealth Scientific and Industrial Research Organisation (CSIRO), Melbourne, Victoria 3169, Australia.

Experimental

General Process of Spray Coating

Spray coating was performed with an ExactaCoat system from Sono-Tek Cooperation equipped with a dual type Impact 120 kHz ultrasonic atomizing nozzle. The nozzle can move in horizontal and vertical direction. The distance between the Impact nozzle and ITO-PEN (13 Ω/□, Peccell Technology, Kanagawa, Japan) substrate is 75 mm. The ultrasonic spray process was carried out at 100 ˚C. During the deposition, nozzle with movable stage was moved in horizontal at a regular speed 5 cm/sec on a fixed substrate with a shadow mask (dimension:10 cm x 1 cm) and then moved down in vertical direction with 1 mm spacing. This movement was repeatedly carried out until we obtained a designed dimension of 10 cm x 1 cm. This is one deposition process. The number of deposition can control a thickness of TiO_2 electrodes. The thickness of electrodes was measured by surface profilometer (Dektak 500, Veeco Instrument INC., Woodbury, NY, USA). These prepared active layer were then transferred to a polyethylene envelope (thickness ~ 80 μm) and sealed under a vacuum of 10^{-1} Torr. Vacuum-sealed TiO_2 films were then pressed at room temperature using a cold isotactic pressure (CIP) instrument (Ilsin Autoclave, South Korea) at 200 Mpa. The thickness of the titania film was dramatically reduced to about 66% of the original thickness after CIP compression at 200 MPa.

Flexible DSC Device Fabrication

DSCs were constructed by attaching a Pt/ITO-PEN to the dye-coated TiO_2/ITO-PEN with an electrolyte composed of 0.04 M I_2, 0.4 M tetrabutylammonium iodide, 0.4 M lithium iodide, 0.3 M *N*-methylbenzimidazole in acetonitrile and 3-methoxypropionitrile by volume 1:1. The electrolyte was filled in between the electrodes by capillary action. All device performance were

measured under open-cell structure without proper sealing like Surlyn between Pt/ITO-PEN and dye-coated TiO_2/ITO-PEN.

Solar cells were tested using simulated sunlight (AM 1.5, 1000 W/m^2) provided by an Oriel solar simulator with an AM 1.5 filter. Current-voltage characteristics were measured using a Keithley 2400 source meter. Cells were biased from high to low, with 10 mV steps and 40 ms settling time.

Incident photon to charge carrier conversion efficiency (IPCE) was conducted with the cell held under short circuit conditions and illuminated by monochromatic light. A cornerstone 260 monochromator was used in conjunction with an optical fibre, Keithly 2400 source meter and 150W Oriel Xe lamp.

I-V Curve

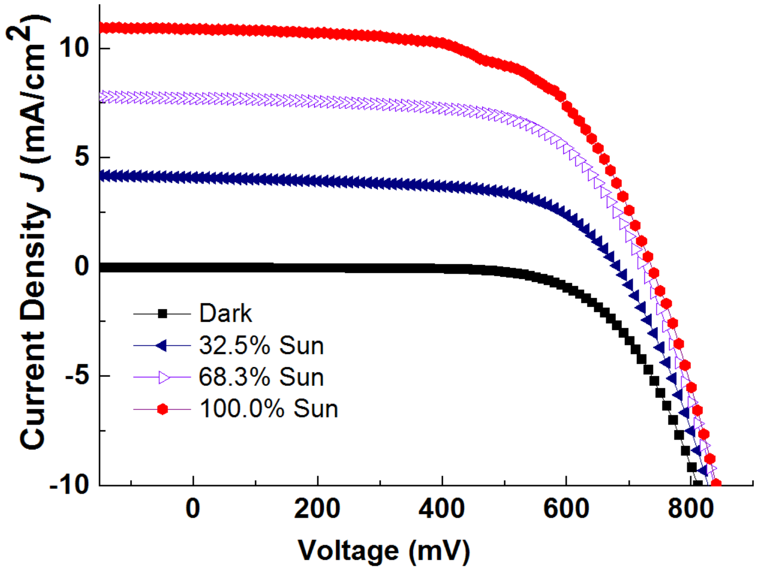

Figure S1. *I-V* curve of a flexible DSC prepared by conventional soaking processes of N719 dye on TiO_2 electrodes prepared by ultrasonic spray technology under various solar simulated light intensities.

Optimum dye:TiO_2 ratio for pre-dye-coated deposition

Various P-25 TiO_2 nanopowder suspensions were prepared, each coated with a different concentration of N719 dye (2.5–10 wt% relative to the weight of TiO_2), so as to determine the optimum dye:TiO_2 ratio. The change in the white color of the P-25 TiO_2 to pink evident in Figure S2 occurred within just a few seconds of the adding N719 dye solution, which is indicative of a near-instant adsorption of the dye molecules. In the case of the 7.5 and 10 wt% suspensions, the supernatants appear dark red in color due to un-adsorbed excess of dye, yet the pink color of the nanopowder particles again indicates complete coverage of their surface with adsorbed dye molecules. However, at 2.5 wt%, the transparent supernatant and vivid pink TiO_2 nanopowder particles indicate that there was insufficient dye to saturate the surface of the TiO_2 nano-particles. Only with a 5.0 wt% suspension were the TiO_2 nano-powder particles homogenously saturated without any dye remaining in the supernatant, which suggests this to be the most suitable concentration for the preparation of pre-dye-coated TiO_2 electrodes. Their device performances are shown.

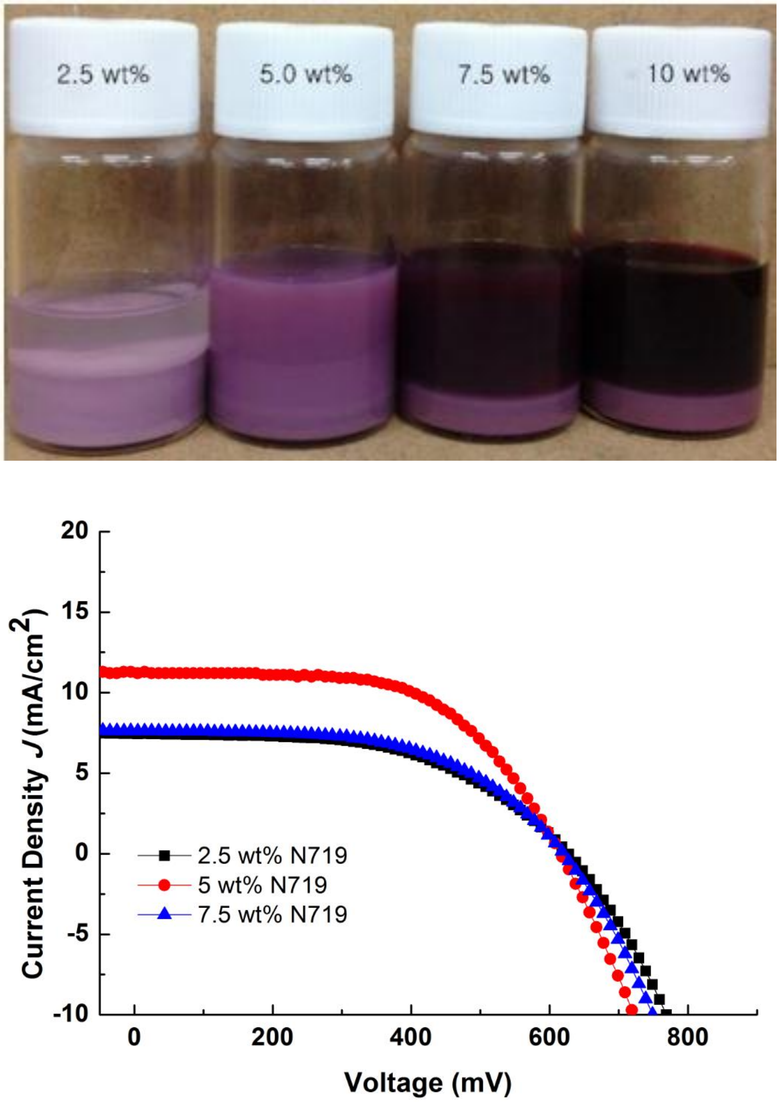

Figure S2. Pre-dye-coated TiO_2 suspensions containing different concentrations of N719 dye (2.5 wt%, 5.0 wt%, 7.5 wt%, and 10 wt%) and corresponding *I-V* curve for 2.5 wt%. 5.0 wt%, and 7.5wt%.

Scanning electron microscopy (SEM)

Figure S3 shows scanning electron microscopy (SEM) surface images of dye-coated TiO_2 samples prepared by pre-dye-coating (a and b), and codeposition (c and d). All SEM images were quite similar. A highly compact structure was obtained for all isostatically pressed films (b and d), unlike for the unpressed films (a and c).

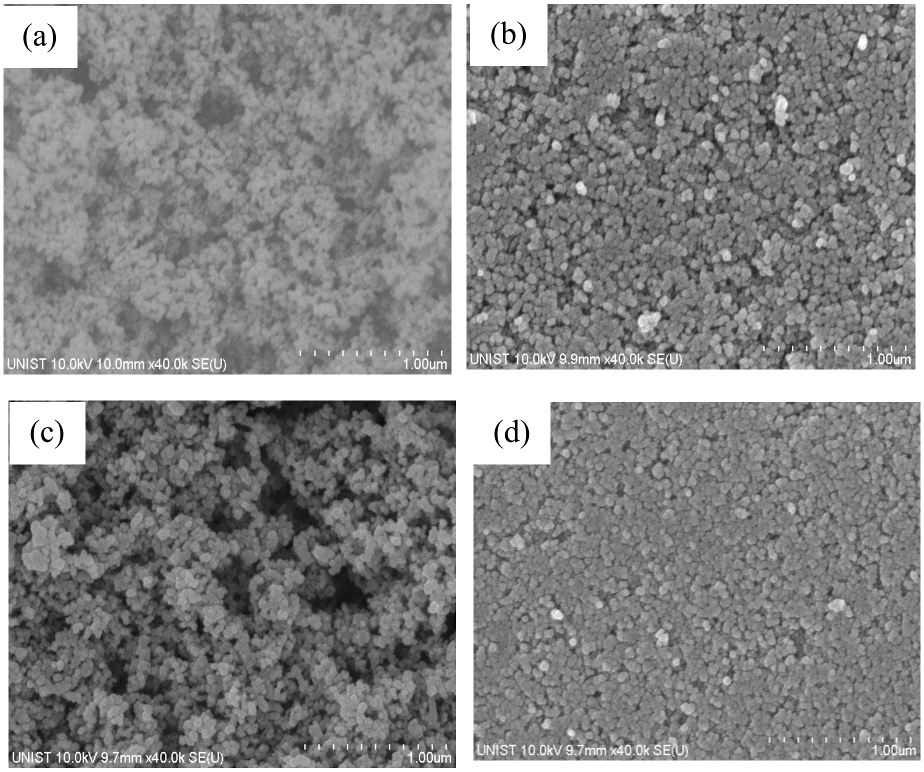

Figure S3. SEM (scanning electron microscopy) images of the surface of N719-coated TiO_2 films prepared using a pre-dye-coating technology (a) before and (b) after cold isostatic pressing, and using codeposition technology (c) before and (d) after cold isostatic pressing.

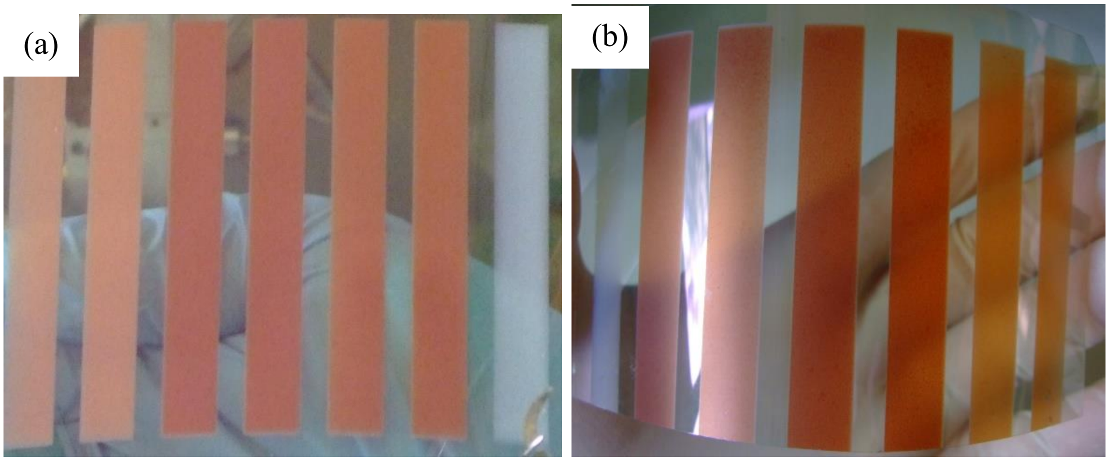

Figure S4. TiO_2 electrodes fabricated by codepoition spray technology with different deposition layers shown (a) before cold isostatic pressing and (b) after cold isostatic pressing.

Table S1. Level of dye attachment (uptake dye) on a TiO2 electrode fabricated by Conventional dye soaking, pre-dye-coating, codepositon method.

method	*Uptake dye* *(mol/cm³)*	*%*
Conventional dye soaking	6.50×10^{-5}	100
Pre-dye-coating	5.71×10^{-5}	88
Codeposition	4.76×10^{-5}	73

Materials Characterization

SEM Image. A field emission scanning electron microscope was used to characterize the morphology of the surface state at before/after isotactic pressure. To acquire the morphology of the surface state at before/after pressure, a field emission scanning electron microscope (Hitachi S-4800) was used.

FT-IR Spectrum. The bonding state of the N719 dye adsorbed on TiO_2 was characterized by fourier transform infrared spectroscopy (Thermo Fisher Scientific Nicolet 6700 FT-IR spectrometer). FT-IR for N719 on TiO_2 (ATR): ν_{COOH} 1720 cm^{-1}, $\nu_{COO^{-}(Symmetric)}$ 1627cm^{-1}, $\nu_{COO^{-}(Asymmetric)}$ 1375cm^{-1}.

UV-Vis Spectrum. UV-Vis spectra were recorded on a commercial spectrophotometer (VARIAN, Australia Pty., ltd). The reference sample was prepared by N719 dyes dissolved in ethanol : water (1:1 v/v %) containing 0.1 M KOH solution. The detached N719 dyes were prepared by the TiO_2 film substrates (size 0.4*0.4 cm^2, thickness 11 μm), which were prepared by pre-dye-coating before press, pre-dye-coating after press, codeposition before press and codeposition process after press, dipping into ethanol : water (1:1 v/v %) containing 0.1M KOH solution about 12 hours.

Raman spectrum. The reference sample was prepared by the TiO_2 film (size 0.4*0.4 cm^2, thickness 11 μm) prepared by ultrasonic spray soaking in 0.5 mM N719 solution about 12 hours. In contrast the pre-dye-coating and codeposition sample were prepared by ultrasonic spray coating technology. Raman spectra were recorded using a Confocal Micro-Raman spectrometer (WITec, Germany) with a 532 nm Ar^+ laser as an excitation source. At 1260, 1475, 1542, 1609 cm^{-1} peaks were obtained due to the vibrational modes of the 4,4'-dicarboxy-2,2'-bipyridine ligand **.**

NMR Spectrum. The reference sample was prepared by N719 dyes dissolved in DMSO-d_6 containing 0.1M NaOD. The detached N719 dyes were prepared by the TiO_2 film (size 0.4*0.4 cm^2, thickness 11 μm), which were prepared by pre-dye-coating along after press and codeposition process after press, dipping into ethanol : water (1:1 v/v %) containing 0.1 M NaOD solution about 2 hours. The desorption solution was evaporated to obtain the solid samples, and then dissolved in DMSO-$d_{6.}$ ^{1}H NMR spectroscopy was carried out with 400 MHz instrument (Agilent 400MHz FT-NMR) . δ 9.20 (2H, d), 8.75 (2H, s), 8.60.

Uptake dye. The uptake dye was derived from the following equation.

$$\frac{concentraiton\ of\ dye\ solution\ (M)}{volume\ of\ dye\ soultuion\ (l)\ \times volume\ of\ TiO_2 electrode\ (cm^3)}$$
